# Structural damage of chicken red blood cells exposed to platinum nanoparticles and cisplatin

**DOI:** 10.1186/1556-276X-9-257

**Published:** 2014-05-23

**Authors:** Marta Kutwin, Ewa Sawosz, Sławomir Jaworski, Natalia Kurantowicz, Barbara Strojny, André Chwalibog

**Affiliations:** 1 Division of Biotechnology and Biochemistry of Nutrition, Faculty of Animal Science, Warsaw University of Life Science, Warsaw 02-786, Poland; 2 Department of Veterinary Clinical and Animal Sciences, University of Copenhagen, Groennegaardsvej 3, Frederiksberg, Copenhagen 1870, Denmark

**Keywords:** Platinum nanoparticles, Cisplatin, Haemolysis, Red blood cells, Cancer therapy

## Abstract

Side effects and resistance of cancer cells to cisplatin are major drawbacks to its application, and recently, the possibility of replacing cisplatin with nanocompounds has been considered. Most chemotherapeutic agents are administered intravenously, and comparisons between the interactions of platinum nanoparticles (NP-Pt) and cisplatin with blood compartments are important for future applications. This study investigated structural damage, cell membrane deformation and haemolysis of chicken embryo red blood cells (RBC) after treatment with cisplatin and NP-Pt. Cisplatin (4 μg/ml) and NP-Pt (2,6 μg/ml), when incubated with chicken embryo RBC, were detrimental to cell structure and induced haemolysis. The level of haemolytic injury was increased after cisplatin and NP-Pt treatments compared to the control group. Treatment with cisplatin caused structural damage to cell membranes and the appearance of keratocytes, while NP-Pt caused cell membrane deformations (discoid shape of cells was lost) and the formation of knizocytes and echinocytes. This work demonstrated that NP-Pt have potential applications in anticancer therapy, but potential toxic side effects must be explored in future preclinical research.

## Background

Cisplatin (*cis*-diamineplatinum(II) dichloride) is a chemotherapeutic drug widely used against various solid tumours and testicular cancer, which has dose-dependent side effects like nephrotoxicity, emetogenesis, ototoxicity, neurotoxicity and haemolysis [1-3]. Repeated intravenous administration of platinum salts may induce haemolytic anaemia by interfering with iron metabolism, decreasing the level of red blood cell precursors and creating an immune-complex between red blood cells (RBC) and cisplatin [4,5]. Moreover, side effects, including haemolytic properties, and resistance of cancer cells to cisplatin are major drawbacks to its application in cancer therapy, and recently, the possibility of replacing platinum salts with nanocompounds has been considered [6-8]. Due to their size (<25 nm), large surface to mass ratio, quantum properties, catalytic activity and high reactivity, platinum nanoparticles (NP-Pt) have unique physicochemical properties and can exert specific effects on a living organism. The bioavailability of NP-Pt colloids depends on particle size, and small NP-Pt (<4 nm) were more barcterio-toxic [9], induced the human cell genotoxic stress (5 to 8 nm) [10] and activated the cell death by apoptosis [11] compared to the bigger nanoparticles (>100 nm) [12,13]. NP-Pt can cause cell cycle arrest and activate apoptosis through the release of Pt^2+^ ions during H_2_O_2_ generation due to the low pH in endosomes [14], and DNA double-strand breaks are caused by Pt^2+^ ions when NP-Pt are incubated with human colon carcinoma cells (HT29) [15]. However, the consequences of *in vivo* NP-Pt administration are not well documented and must be elucidated in future research [9].

Haemolytic properties and interactions with RBC are main parameters for the biocompatibility of nanoparticles [5,16]. The bloodstream is the main translocation path for glucose, oxygen and carbon dioxide, as well as nanoparticles. Incubation of silver (Ag), gold (Au) and Pt nanoparticles with human RBC demonstrated that Au and Pt nanoparticles are non-haemolytic but cause haemagglutination, while Ag nanoparticles are haemolytic [14]. *In vitro* haemocompatibility studies indicated that biosynthesised Pt nanoparticles had negligible haemolytic effects [17], while Ashranani et al. reported that Pt and Au nanoparticles (compared to Ag) were more haemocompatible and did not show the agglutination of erythrocyte and also no precipitation properties [14]. On the other hand, Ag nanoparticles did not cause significant changes in micronucleated polychromic erythrocytes, and no dose-dependent differences in haemolysis were observed [3]. Recent studies have demonstrated the haemolytic properties of other nanoparticles like polystyrene [18], PLGA [4], TiO_2_[19] and mesoporous silica [20].

This study evaluated the hypothesis that NP-Pt may affect the morphology of chicken embryo RBC, causing haemolysis and structural damage comparable to Pt salt. The objective of this preliminary work was to compare the biocompatibility of NP-Pt and cisplatin with chicken embryo RBC.

## Methods

### Hydrocolloids

**Figure 1:**
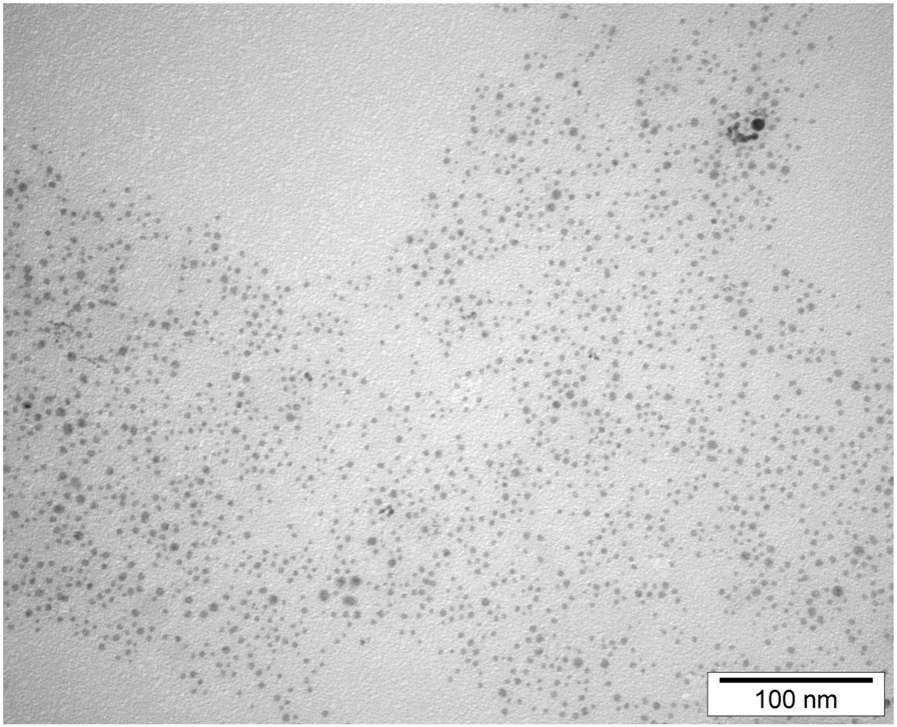
**TEM imagines of platinum nanoparticles.** Scale bar, 100 nm.

NP-Pt hydrocolloid was obtained from Nano-koloid (Warsaw, Poland), which was produced by a patented electric non-explosive method (Polish patent 380649) from high-purity metal (99.9999%) and high-purity demineralized water, and diluted to 2.6 μg/ml in phosphate buffered saline (PBS: pH 7.2; P4417, Sigma-Aldrich, St. Louis, MO, USA).The morphology of NP-Pt was inspected using a JEOL JEM-1220 transmission electron microscope (TEM, JEOL Ltd., Tokyo, Japan) at 80 KeV equipped with a Morada 11 megapixel camera (Olympus Corporation, Tokyo, Japan) (Figure 1). Triplicate samples of NP-Pt were prepared for TEM by placing droplets of the hydrocolloid onto Formvar-coated copper grids (Agar Scientific Ltd., Stansted, UK) and air drying before TEM imaging (Figure 1). The zeta potential of the NP-Pt hydrocolloid was measured by the electrophoretic light-scattering method using a Zetasizer Nano-ZS90 (Malvern, Worcestershire, UK). Each sample (20 replicates) of NP-Pt was measured after 120 s of stabilization at 25°C.

Cisplatin (*cis*-diamineplatinum(II) dichloride) was obtain from Sigma (479306; Sigma, St. Louis, MO, USA) and diluted to 4 μg/ml in PBS immediately before use. The final concentration of cisplatin hydrocolloid was 4 μg/ml of cisplatin, because of the potential and the best efficiency (67% of mice tumour free after 4 μg/ml cisplatin treatment) for intratumoural injection [1]. The concentration of NP-Pt (2.6 μg/ml) was equal to the atomic mass of Pt atoms in cisplatin.

### Embryo model

Fertilized eggs (*n* = 20; 56 ± 2.2 g) from Ross Line hens were obtained from a commercial hatchery, stored at 12°C for 4 days and incubated under standard conditions for an additional 19 days (temperature 37.5°C, 60% humidity, turning once per hour). On day 20, the embryos were sacrificed by decapitation and blood samples (one sample from each embryo) collected from the jugular vein. Blood samples were divided into four groups: control untreated (0% haemolysis), positive control treated with 3% hydrogen peroxide (100% haemolysis), 4 μg/ml cisplatin [1] and 2.6 μg/ml NP-Pt hydrocolloid diluted in PBS. The samples were placed in Vacutainer tubes (BD Inc., Franklin Lakes, NJ, USA) containing ethylenediaminetetraacetic acid (EDTA), gently mixed on a rotary shaker and incubated for 3 h at 37°C. The incubation time was based on the research publications [14,21] and an adjustment of the standard ASTM F-756-00 [22].

All measurements were performed in three repetitions (each experimental group in triplicate).

### Blood cell morphology

#### ***Light microscopy***

Peripheral blood smears were made using 5 μl of blood, air-dried and stained by May-Grünwald-Giemsa. Peripheral blood smears were examined at × 1,000 magnification (Leica DM750, Leica Microsystems, Nussloch, Germany).

#### ***Scanning electron microscopy***

Scanning electron microscopy (SEM) analysis of RBC was performed by means of an FEI Quanta 200 electron microscope (FEI Co., Hillsboro, OR, USA). The blood samples were rinsed in PBS (0.01 M, pH 7.2; P4417, Sigma) then fixed in 2.5% glutaraldehyde (G5882, Sigma) for 1 h, washed twice in 0.1 M PBS (0,01 M, pH 7.2; P4417, Sigma) and placed on aluminium SEM stubs. The SEM stubs were kept in a moist atmosphere for 1 h, washed in PBS (0.01 M, pH 7.2; P4417, Sigma), post-fixed in 1% osmium tetroxide (75632, Sigma) for 1 h, rinsed in distilled water and dehydrated in graded ethanols. After critical point drying with liquid CO_2_ in a vacuum apparatus (Polaron CPD 7501, Quorum Technologies, Newhaven, East Sussex, UK) and coating with gold-palladium (JEE-4C, JEOL Ltd., Tokyo, Japan), the samples were inspected by SEM at 1 KeV (FEI Quanta 200).

#### ***Haemolytic assay***

The haemolysis assay was performed with chicken whole blood. After incubation, the tubes containing blood samples from the groups - control untreated (0% haemolysis), positive control treated with 3% hydrogen peroxide (100% haemolysis), 4 μg/ml cisplatin [1] and 2.6 μg/ml NP-Pt hydrocolloid - were centrifuged at 1,200 rpm (Sorvall ST 16, Thermo Fisher Scientific, Waltham, USA) for 10 min to collect the plasma. The supernatant was analysed for the presence of the haemoglobin at 540 nm (Infinite M200, Tecan, Durham, NC, USA) and percent haemolysis calculated according to Shiny et al. [17].

#### ***Statistical analysis***

The data was evaluated by mono-factorial analysis of variance (ANOVA). The differences between groups were tested by the multiple-range Tukey test using Statgraphic Centurion ver. XV (Statpoint Technologies, Warrenton, VO, USA). Differences with *P* < 0.05 were considered significant.

## Results

### Nanoparticles

TEM observation of NP-Pt did show that NP-Pt had regular spherical shape ranging from 2 to 19 nm, and also the aggregation of nanoparticles was not observed (Figure 1). The mean zeta potential of the NP-Pt was −9.6 mV.

### Evaluation of morphological changes by microscopy

**Figure 2:**
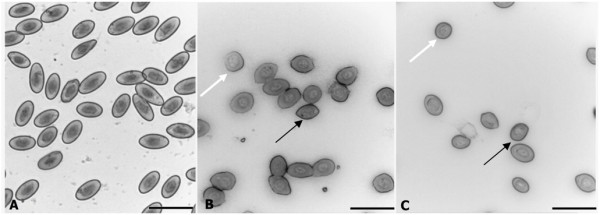
**RBC morphology by light microscopy. (A)** Control (without treatment), **(B)** cisplatin and **(C)** NP-Pt. Black arrow, deformation of erythrocytes; white arrow, swollen RBC; g, ghost cells. Scale bar, 20 μm.

**Figure 3:**
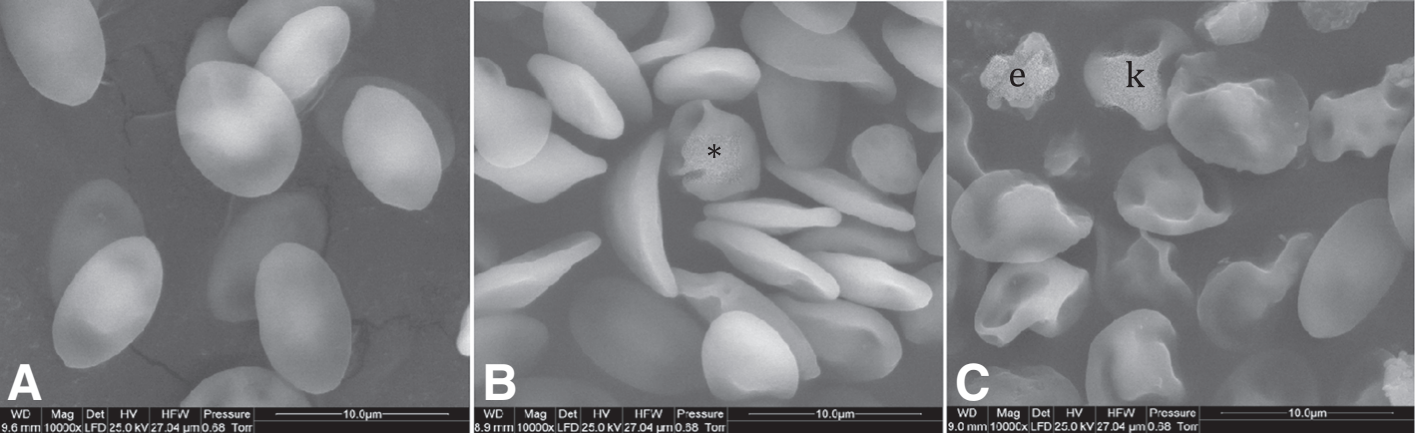
**Visualization of RBC morphology by scanning electron microscopy. (A)** Control (without treatment), **(B)** cisplatin and **(C)** NP-Pt. Asterisk, keratocyte; k, knizocyte; e, echinocyte. Scale bar, 10 μm.

Evaluation of RBC by light microscopy demonstrated that NP-Pt and cisplatin had harmful effects on morphology. The cell membranes were damaged, the shape of cells was deformed and cells lost their biconcavity. Observation also showed an increasing level of swollen cells as a result of membrane damage after osmotic pressure on red blood cells (Figure 2). Exposure of RBC to NP-Pt and cisplatin caused haemagglutination. Scanning electron microscopy revealed ruffled cells, membrane deformations and loss of the discoid shape of RBC treated with NP-Pt. After cisplatin treatment, keratocytes were seen (Figure 3B), indicating that cisplatin had haemolytic properties. After NP-Pt treatment (Figure 3C), there were structural changes in the RBC, and knizocytes were also seen. SEM analysis of RBC treated with NP-Pt also showed early echinocytes with blebs and protuberances on their surfaces.

### Haemolysis

**Figure 4:**
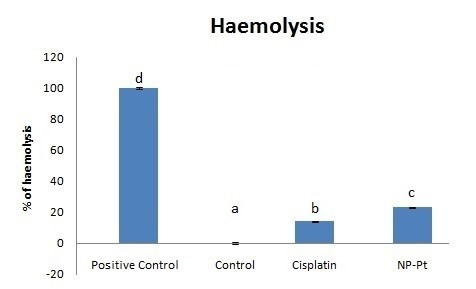
**Haemolysis of RBC.** Bars with different superscripts denote a statistically significant difference between the control group (untreated) and groups treated with the following: 3% hydrogen peroxide (positive control), cisplatin (4 μg/ml) or NP-Pt (2.6 μg/ml). Error bars are standard error of the mean. The bars with different letters indicate significant differences, *P* < 0.05.

NP-Pt and cisplatin were haemolytic compared to the control group (Figure 4), and the percent haemolysis was higher for NP-Pt (23%) than for cisplatin (14%). The lack of haemolysis in the control group and almost 100% haemolysis in the positive control group (treated with 3% hydrogen peroxide) confirmed the accuracy of the assay.

## Discussion

Chicken embryo RBC were exposed to NP-Pt and the antineoplastic drug cisplatin, which allowed haemocompatibility to be evaluated in a short time, as well as serving as a fast, precise model for evaluating toxicity [11,23,24]. It has been demonstrated that NP-Pt had harmful effects on *in vivo* mice model, causing kidney injury after intravenous administration [25] and inflammatory responses after intratracheal instillation [26]. Furthermore, NP-Pt had harmful effect on *in ovo* model, causing mitochondria degradation at chicken embryo brain tissue ultrastructure, activation of apoptosis and reduced proliferation rate of the brain cells [11]. In the present research, NP-Pt at 2.6 μg/ml incubated with whole chicken blood affected RBC morphology and haemocompatibility status. Comparing the effects of NP-Pt and cisplatin on RBC structures, there were substantial differences between NP-Pt and cisplatin. The RBC treated with NP-Pt lost their biconcavity and showed corrugations and damage to cell membranes. The analysis of peripheral blood smears from the NP-Pt group revealed the presence of ghost cells, which are the result of cell lysis and release of haemoglobin from RBC [27]. On the other hand, other studies did not show any harmful effect on human RBC structure [21].

The haemolytic properties of Pt drugs during chemotherapy are also responsible for higher risks of blood diseases like anaemia [28]. Comparing the results of this study with previous reports on the haemocompatibility of cisplatin confirmed the haemolytic activity of Pt-based drugs as one of the main side effects causing haemolytic anaemia and also bone marrow diseases and haemolysis [2,29]. The present results demonstrated that both cisplatin and NP-Pt induced haemolysis compared to the control group. Evaluation of haemocompatibility of nanoparticles should be considered as one of the factors of assessing systemic toxicity. Haemolytic properties of the nanoparticles can be determined by spectrophotometric measurement (540 nm) and based on adjustments of the ASTM F-756-00 standard [22]. Human RBC have different morphology than chicken RBC, with the primary difference being that chicken RBC have a nucleus containing genetic material. The mechanism of action for platinum-containing anticancer drug utilizes the presence of DNA to induce cancer cell death by apoptosis [30,31]. Regarding the antineoplastic properties of ciplatin, it is important to emphasize that the intravenous administration of cisplatin has severe side effect on blood compartments. The present results confirmed that cisplatin and NP-Pt not only induced haemolysis but also caused the structural damage of RBC. Haemolytic as well as toxic properties of nanoparticles can be reduced by increasing the particle size [12] or by excluding intravenous administration.

Haemocompatibility studies of Pt, Au and Ag nanoparticles demonstrated that only Ag has harmful effects on the structure of human RBC and significantly affected haemolytic status [14]. On the other hand, NP-Pt were described as haemocompatible materials [21]. Furthermore, interactions between NP-Pt and human RBC indicate that NP-Pt have minor effects on cell membrane structure but still cause haemolysis [17]. The present results revealed that cisplatin treatment led to formation of keratocytes, indicating that cisplatin was haemolytic, which may be due to mechanical fragility or oxidant injury to RBC [32]. The results also showed that NP-Pt treatment caused the damage of RBC and also appearance of knizocytes. Knizocytes are RBC with multiple concavities due to indentations of the cell membrane, observed in newborn mammals as a young type of RBC with cell membrane deformations, and also their appearance indicates an impaired microcirculation or impaired oxygen-carrying capacity [33] SEM analysis revealed that after NP-Pt treatment, early echinocytes were observed which may indicate harmful effects to RBC and a side effect often associated with haemolytic anaemia [32,33].

The present results indicated that the percent haemolysis after NP-Pt treatment was higher compared to cisplatin treatment. These finding may indicate that NP-Pt were toxic to RBC by direct interactions with 2.4 pg of genetic material in chicken RBC [34]. Furthermore, chicken nuclear DNA has a 42% to 43% guanine and cytosine base-pair content [35] which is acted upon by cisplatin, where guanine bases (especially the N7 sites) are preferred sites for Pt binding to DNA [36]. Damage of RBC under the influence of NP-Pt probably was related to the ability to arrest the cell cycle and also the cytotoxicity of NP-Pt [6,10,37]. These mechanisms may be responsible for the pathomorphological changes in chicken RBC and also for a high occurrence of haemolysis.

## Conclusions

NP-Pt and cisplatin incubated with chicken embryo RBC caused damage to the structure of RBC and induced haemolysis. Treatment with cisplatin caused structural damage to cell membranes and the appearance of keratocytes, while NP-Pt caused cell membrane deformation, loss of the discoid shape and the formation of knizocytes and echinocytes. These preliminary results indicated that NP-Pt could be utilized for cancer therapy instead of cisplatin, but potential side effects must be evaluated in future research.

## Abbreviations

ANOVA: analysis of variance; cisplatin: *cis*-dichlorodiammineplatinum(II); EDTA: ethylenediaminetetraacetic acid; NP-Pt: nanoparticles of platinum; PBS: phosphate buffered saline; RBC: Red blood cells; SEM: scanning electron microscope; TEM: transmission electron microscope.

## Competing interests

The authors declare that they have no competing interests.

## Authors' contributions

MK carried out RBC studies and drafted the manuscript. ES conceived the study and helped draft the manuscript. SJ participated in the design of the experiment. NK participated in the statistical analysis. BS participated in the SEM analysis. AC participated in the design and coordination and helped draft the manuscript. All authors read and approved the final manuscript.

## Authors' information

MK, SJ, NK and BS are PhD students at the Warsaw University of Life Sciences (WULS). ES is a PhD, DSc, professor and head of department at WULS. AC is a DSc, professor and head of division at the University of Copenhagen (UC).
